# Cytotoxic and antimicrobial activity of selected Cameroonian edible plants

**DOI:** 10.1186/1472-6882-13-78

**Published:** 2013-04-08

**Authors:** Jean Paul Dzoyem, Santosh Kumar Guru, Constant Anatole Pieme, Victor Kuete, Akash Sharma, Inshad Ali Khan, Ajit Kumar Saxena, Ram Anuj Vishwakarma

**Affiliations:** 1 Department of Biochemistry; Faculty of Science, University of Dschang, Dschang, Cameroon; 2 Cancer Pharmacology Division, Indian Institute of Integrative Medicine, Jammu, India; 3 Department of Physiological Sciences and Biochemistry, Faculty of Medicine and Biomedical Sciences, Yaounde, Cameroon; 4 Clinical Microbiology Division, Indian Institute of Integrative Medicine, Jammu, India; 5 Medicinal Chemistry Division, Indian Institute of Integrative Medicine, Jammu, India

**Keywords:** Cytotoxic, Antimicrobial, Edible plants, Cameroon

## Abstract

**Background:**

In Cameroon, the use of edible plants is an integral part of dietary behavior. However, evidence of the antimicrobial as well as the cytotoxic effects of many of them has not been investigated. In the present study, aqueous and methanol extracts from barks, seeds, leaves and roots of three Cameroonian edible plants namely *Garcina lucida, Fagara heitzii* and *Hymenocardia lyrata* were evaluated for their cytotoxic and antimicrobial activities.

**Methods:**

Antibacterial and antifungal activities were assessed by the broth micro-dilution method meanwhile the cytotoxicity was performed using sulphorhodamine B assay (SRB) against the human leukemia THP-1, the alveolar epithelial A549, prostate cancer PC-3, breast adenocarcinoma MCF-7 and cervical cancer HeLa cell lines.

**Results:**

The minimum inhibitory concentration (MIC) values of the seven tested extracts ranged from 62.5 μg/ml to 1000 μg/ml. The methanol (MeOH) extract from the roots of *H. lyrata* showed the highest antibacterial activity against Gram-positive bacteria *S. aureus* and *S. epidermitis.* The best antifungal activity was obtained with the MeOH extract from the leaves of *G. lucida* against *C. tropicalis* (MIC value of 62.5 μg/ml). The *in vitro* antiproliferative activity revealed that, extract from the bark of *F. heitzii* and extract from *H. lyrata* roots had significant cytotoxic activity on THP-1 (IC_50_ 8.4 μg/ml) and PC-3 (IC_50_ 9.5 μg/ml) respectively.

**Conclusion:**

Our findings suggest that Cameroonian spices herein studied could be potentially useful for the development of therapeutic agents against bacterial infections as well as for prostate and leukemia cancer.

## Background

Plants have served humans well as valuable components of seasonings as well as medicines, and have played a significant role in maintaining human health and improving the quality of human life for thousands of years. There is no doubt that increasing our intake of spices is one of the most effective, convenient and economical ways in which we can fortify ourselves against infectious diseases and related cancers [1]. In the area of cancer prevention, plants consumption such as spices and their constituents as potential chemopreventive agents remains an extensive research topic. Numerous studies have been published in regards to the relation between plants consumption, cancer prevention, antimicrobial effects, and overall protection of human health [2]. In Cameroon, medicinal plants consumption is an integral part of dietary behavior, but relatively little is known about their antimicrobial potential and anti-cancer effects. The three selected edible plants studied herein, namely *Garcinia lucida* Vesque*, Fagara heitzii* (Guill and Perr) Engl. and *Hymenocardia lyrata* Tul are recognized for their medicinal virtues and have been reported to possess various biological activities. The barks, seeds and leaves of *G. lucida* are used to treat gastric infections, gynecological infections, diarrheas and as anti-poison [3,4]. It has been reported to have antileishmanial and anti-trypanosomal properties [5,6]. Seeds and barks of *Fagara heitzii* (Guill and Perr) Engl have been used to treat abdominal pains, asthma, appendicitis and toothache [7]. *H. lyrata* is well known in Cameroon, where roots and barks are used as antiparasitic, against gastric ulcer and in high blood pressure regulation [8]. There are few numbers of reports about the potential of Cameroonian edible plants in terms of preventing and treating microbial diseases and cancer [9,10]. The objective of this study was to evaluate aqueous and methanol extracts from barks, seeds, leaves and roots of three Cameroonian plants edible plants for their anticancer activity on human cancer cell lines of various tissues including as cervix, leukemia, prostate, lung and breast; as well as their antimicrobial activities against bacterial and fungal pathogens.

## Methods

### Plant material and extraction

*F. heitzii (*Rutaceae), *G. lucida* (Guttiferae) and *H. lyrata* (Euphorbiaceae)were harvested in the East region, Ebolowa and Batchingou in Cameroon. The plants were identified at the Cameroon National Herbarium where voucher specimens were deposited under the reference number 1441/HNC, 53354/HNC and 32301/HNC respectively for *F. heitzi, G. lucida and H. lyrata.*

Each collected sample (leaves and barks for *G. lucida*, roots and barks of *H. lyrata,* and fruits and roots for *F. heitzii*) was dried at room temperature (28 ± 3°C), pulverized and powdered. Each powder (50 g) was macerated in 500 ml of methanol or water for 72 h at room temperature. After 72 h, the mixture was filtered using a paper filter Whatman No. 1. Each filtrate was then concentrated under vacuum (Rotary evaporator, Heidolph WB 200) to obtain the crude extract. Each crude extract obtained was then weighed and stored at 4°C.

### Cytotoxic activity assay

#### Cell lines and treatment

The effect of the extracts and compounds on cell growth was determined in a panel of human tumor cells including lung A549 adenocarcinoma, breast carcinoma MCF-7, prostate carcinoma PC-3, cervical carcinoma HeLa and acute monocytic leukemia cell line THP-1, obtained from National Cancer Institute, USA. THP-1, A-549 and PC-3 were maintained in RPMI medium while MCF-7 and HeLa were cultured in MEM medium. All media used were supplemented with 10% fetal bovine serum (FBS), 100 IU/ml penicillin. The cell lines were maintained under standard cell culture conditions at 37°C and 5% CO_2_ in a humidified environment.

#### Cytotoxic activity by SRB assay

*In vitro* cytotoxicity against above mentioned five human cancer cell lines was determined using sulphorhodamine B assay (SRB) as described previously [11]. Briefly, cells were harvested in log phase using trypsin (0.05% trypsin, 0.02% EDTA, in PBS). The cell suspensions were diluted with appropriate growth medium to obtain the cell densities depending on the cell line: (10^4^ cells/well for HeLa, 10^4^ cells/well for A549, 10^4^ cells/well for THP-1, 1, 5×10^4^ cells/well for MCF-7 and 10^4^ cells/well for PC-3). An aliquot of 100 μl of each suspension were seeded in 96 wells cell culture plates. The cells were incubated at 37°C in an atmosphere of 5% CO_2_ and 95% relative humidity in a CO_2_ incubator. After 24 h incubation, test materials (100 μl/well) at varying concentrations (1, 10, 30 and 50 or 100 μg/ml) were added to the wells containing cells. Paclitaxel 0.1, 1 and 10 μM was used as positive control. Suitable controls with equivalent concentration of DMSO were also included. The plates were further incubated for 48 h in a CO_2_ incubator after addition of test material. After incubation cells were fixed by gently layering trichloroacetic acid (50 μl/well, 50% w/v) on top of the medium in all the wells and incubated at 4°C for 1 h. The plates were washed five times with distilled water and air-dried. Cell growth was measured by staining with sulforhodamine B dye (0.4% w/v in 1% acetic acid, 100 μl/well). The unbound dye was washed 3–5 times with 1% acetic acid and plates were air dried. The adsorbed dye was dissolved in Tris-Buffer (100 μl/well, 0.01 M, pH 10.4) and plates were gently shaken for 10 min on a mechanical shaker. The optical density (OD) was recorded using a 96 well plate reader. Growth inhibition was calculated by subtracting mean OD values of respective blank from the mean OD value of experimental set. Percentage growth in presence of test material was calculated considering the growth in absence of any test material as 100% and in turn percentage growth inhibition in presence of test material was calculated. The viability and growth in the presence of test material is calculated by following formula.

$$\begin{array}{l} \mathrm{Growth}\;\mathrm{inhibition}\;\left(\%\right)=\left[OD_{\left(\mathrm{test}\;\mathrm{sample}\right)}-OD_{\left(\mathrm{blank}\right)}/\right)\\ \;\left(OD_{\left(\mathrm{control}\right)}-OD_{\left(\mathrm{blank}\right)}\right]\times 100 \end{array}$$

IC_50_ value is the concentration of sample required to inhibit 50% of the cell proliferation and was calculated by plotting the percentage survival versus the concentrations, using Microsoft Excel. For all samples, each compound concentration was tested in triplicates in a single experiment.

### Antimicrobial assays

#### Microbial growth conditions

A total of ten microbial strains were tested obtained from the American Type Culture Collection for their susceptibility to extracts and compounds. These strains comprised of three yeasts: *Candida albicans* (ATCC 90028), *Candida krusei* (ATCC 6258), and *Candida tropicalis* (ATCC 750); one filamentous fungi: *Aspergillus fumigatus* (MTCC 1811); three Gram-negative bacteria: *Pseudomonas aeruginosa* ATCC 27853, *Escherichia coli* ATCC25292, vancomycin-resistant *Enterococcus faecalis* (VRE) and three Gram-positive bacteria: *Staphylococcus aureus* ATCC 29213, methicillin-resistant *Staphylococcus aureus* (MRSA, ATCC 33591) and *Staphylococcus epidermidis* (ATCC 12228). They were maintained on agar slant at 4°C and sub-cultured on a fresh appropriate agar plates 24 h prior to any antimicrobial test. The Mueller Hinton Agar (MHA) and Sabouraud dextrose Agar (SDA) were used for the activation of bacteria and fungi respectively. The Mueller Hinton Broth (MHB) and RPMI 1640 were used for the MIC determinations.

#### Inoculum preparation

Suspensions of bacteria and yeasts were prepared in sterile normal saline (0.85%) from 24 h grown on SDA or MHA at 37°C. The turbidity of the microbial suspension was adjusted with a densitometer to a McFarland standard of 0.5 for bacteria and 0.9 for yeast, which are equivalent to 1–5 × 10^8^ CFU/ml and 1–5 × 10^7^ CFU/ml respectively.

Inoculum suspensions of *Aspergillus* species were prepared from fresh, mature (3 to 5 days old) cultures grown on Sabouraud agar or potato dextrose agar slants. The colonies were covered with approximately 5 ml of distilled containing 5% Tween 20. Then, the suspensions were made by gently probing the colony with the tip of a Pasteur pipette and transferred to a sterile tube; the resulting suspensions were homogenized for 15 s with a vortex mixer at 2000 rpm. The suspension was filtered and collected in a sterile tube. The inoculum size was adjusted to 1-5 × 10^6^ spores/ml by microscopic enumeration with a cell-counting hematocytometer. Adjusted suspensions were checked by plating 0.01 ml of a 1:100 dilution onto PDA plates to determine the viable number of CFU/ml. The plates were incubated at 37°C and observed daily for the presence of fungal colonies. The colonies were counted as soon as possible after the observation of visible growth.

#### MIC determination

The MIC was performed by broth microdilution method, with Mueller Hinton Broth (MHB) for bacteria and RPMI 1640 medium (containing L-glutamine, without sodium bicarbonate and buffered to pH 7.0 with 0.165 M morpholine propanesulfonic acid) for fungi. Stock solutions of extracts were prepared in 100% dimethylsulfoxide (DMSO; Sigma) and twofold serial dilutions were prepared in media in amounts of 100 μl per well in 96-well. The above-mentioned microbial suspensions were further diluted to 1:100 in media, and a 100 μl volume of this diluted inoculum was added to each well of the plate, resulting in a final inoculum of 1.5×10^6^ cfu/ml for bacteria, 1.5×10^4^ cfu/ml for *A. fumigatus* and 1.5×10^5^ cfu/ml for yeasts. The final concentration of samples ranged from 7.8-1000 μg/ml. The medium without the agents was used as a growth control and the blank control used contained only the medium. Ciprofloxacin and Amphotericin B served as the standard drug controls. The microtiter plates were incubated at 37°C for 24 h, 48 h and 72 h respectively for bacteria, yeasts and *Aspergillus* species. The plates were read visually, and the MIC was defined as the lowest concentration of the antifungal agents that prevented visible growth with respect to the growth control.

### Statistical analysis

The one-way ANOVA at 95% confidence level was used for statistical analysis.

## Results and discussion

**Figure 1:**
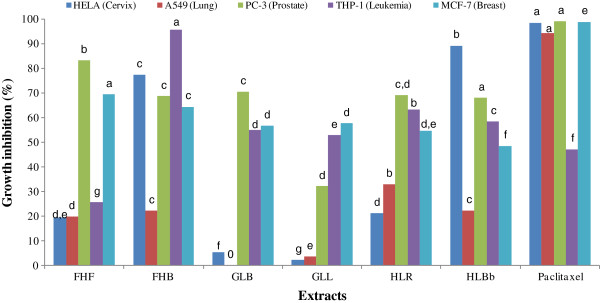
**Cytotoxic activity of Extracts and paclitaxel determined by the percentage of growth inhibition.** Extracts were tested at 100 μg/mL. Data with different alphabetic letters are significantly different (P < 0.05). FHF: Methanol extract from *Fagara heitzii* fruits; FHB: Methanol extract from *Fagara heitzii* barks; GLB: Methanol extract from *Garcina lucida* barks; GLL: Methanol extract from *Garcina lucida* leaves; HLR: Methanol extract from *Hymenocardia lyrata* roots; HLBb: Methanol extract from *Hymenocardia lyrata* barks.

In the present study, we have evaluated the antimicrobial and antiproliferative activity of MeOH and aqueous extracts from three edible plants used in Cameroon. In a preliminary screening, the antiproliferative activity of extracts was assessed on a panel of five human cancer cell lines at a single concentration of 100 μg/ml. The five human cell lines used were representative of tumors from a five types of human tissue including blood, lung, breast, prostate and cervix tissues. Result of the growth inhibition effects are shown in Figure 1. A perusal of this figure revealed that all the extracts tested induced more than 50% cell death on at least one of the five cell lines. The most potent extract identified, the methanol extract from *Fagara heitzii* barks (FHB) were found to induce over 60% cell death in 4 out of 5 cell lines. With the respective highest growth inhibition percentage of 95.55%, 69.30% and 83.11%, the acute monocytic leukemia cell line THP-1, breast carcinoma MCF-7 and cervical carcinoma HeLa) were more sensitive to the extracts than the two others tumor cell lines tested (lung A549 adenocarcinoma, prostate carcinoma PC-3). This observation indicated that the extracts selectively inhibited the growth of different tumor cells. To some extent, these results were similar to those of previous studies that extracts from some spices selectively inhibit the growth of human cancer cell [10,12,13]. In contrast, all the extracts had low inhibitory effects on the growth of A549 suggesting the resistance of this cell line towards the tested samples.

All the extracts were initially screened at one concentration (100 μg/ml); then dose-dependant inhibition was further performed to determine IC_50_ (Table 1). Results indicated that almost all plant extracts exerted cytotoxic activity against at least one of the five cell lines used (IC_50_ varying from 8.4 μg/ml to 99 μg/ml). Significant antiproliferative properties were observed with FHB and the methanol extract from *H. lyrata* roots (HLR) with IC_50_ of 8.4 μg/ml and 9.5 μg/ml against THP-1 and PC-3 respectively. Interesting cytotoxic activity was also observed against MCF-7 cell line, for the methanol extract from *F. heitzii* fruits and barks (FHF and FHB respectively) and the methanol extract from *H. lyrata* roots (HLR) with IC_50_ of 26 μg/ml, 42 μg/ml and 32 μg/ml respectively, while extract from *Garcinia lucida barks and leaves* (GLB and GLL respectively) demonstrated weak antiproliferative ability with IC_50_ of 73 μg/ml and 82 μg/ml respectively. According to the criteria of the American National Cancer Institute the anticancer activity of a crude extract promising for further purification based in the IC_50_ values is lower than 30 μg/mL [14]. Considering this criteria, IC_50_ values of FHF (IC_50_ of 26 μg/ml), FHB (IC_50_. of 8.4 μg/ml) and HLR (IC_50_. of 9.5 μg/ml) respectively on MCF-7, THP-1 and PC-3 are well within the limit. Therefore, FHF, FHB and HLR could be considered as promising sources for new natural products with cytotoxic properties. Extracts from *F. lepreuiri*, *Fagara macrophylla* and *Garcinia lucida* (fruits) have earlier been reported to contain potentially antiproliferative activity [10]. These results are consistent with previous work that has been carried out on dietary plants confirming their ability to prevent cancer [2].

**Table 1:** **IC**_**50 **_**values of the extracts from three Cameroonian edible plants and paclitaxel against human cancer cell lines**

**Extracts**	**Cell lines and IC**_**50 **_**(μg/mL)**				
	**HeLa (Cervix)**	**A549 (Lung)**	**PC-3 (Prostate)**	**THP-1 (Leukemia)**	**MCF-7 (Breast)**
FHF	-	-	56	-	26
FHB	66	-	76	8.4	42
GLB	-	-	61	72	73
GLL	-	-	-	38	82
HLR	99	-	9.5	36	32
HLBa	nd	nd	nd	nd	nd
HLBb	44	-	68	64.5	-
Paclitaxel	12	1	62	4	3

FHF: Methanol extract from *Fagara heitzii* fruits; FHB: Methanol extract from *Fagara heitzii* barks; GLB: Methanol extract from *Garcina lucida* barks; GLL: Methanol extract from *Garcina lucida* leaves; HLR: Methanol extract from *Hymenocardia lyrata* roots; HLBa: Aqueous extract from *Hymenocardia lyrata* barks; HLBb: Methanol extract from *Hymenocardia lyrata* barks; -: value above 100 μg/mL; nd: not determined.

Results of the antimicrobial assay are depicted in Table 2. All the extracts tested in this study exhibited antimicrobial activities against bacteria and/or fungi. The minimum inhibitory concentration (MIC) values ranged from 62.5 μg/ml to 1000 μg/ml. HLR had the highest antibacterial and antifungal activity against Gram-positive bacteria *S. aureus* and *S. epidermitis*. According to Kuete [15], the antimicrobial activity of extracts can be classified as follows: significant if MIC values are below 100 μg/ml, moderate when 100<MIC<625 μg/ml and weak if MIC>625 μg/ml. Therefore, the overall antibacterial activity exhibited in this study varied from weak to significant. Among the six bacterial strains tested, the two Gram-positive bacteria (*S. aureus* and *S. epidermitis*) were the most sensitive to the extracts, while the two Gram-negative bacteria strains (*E. coli* and *P. aeruginosa*) were the most resistant. These results were consistent with the common observation that Gram-positive bacteria are generally more sensitive to the spice and herb extracts than Gram-negative bacteria. The resistance of Gram-negative bacteria towards antibacterial substances is due to their outer membrane that contributes to the intrinsic resistance by acting as an efficient permeability barrier [16]. The antibacterial activity of other edible plants was previously reported by many authors [17,18]. It is noteworthy that HLR showed moderate growth inhibiting activity against *MRSA* and *VRE* which are two bacterial strains expressing MDR phenotypes. Probably the activity exerted by these extracts is due to the presence of natural bioactive compounds with either a new mode of action or which are able to escape or to inhibit the mechanism of resistance of these MDR bacteria. Previous study reported the ability of some plant extracts to inhibit efflux pumps resistance mechanism in bacteria [9,19]. The tested extracts differed greatly in their activity against fungi and the best inhibition was observed with GLL with MIC of 62.5 μg/ml against *C. tropicalis*. In a similar study, Hamza et al. [20] reported that extracts having MIC of 0.5 mg/ml or less as being strong inhibitors of fungal growth. Their report was based on classification of MIC earlier proposed by Aligiannis et al. [21] who classified plant extracts having MIC of 0.5 mg/ml as strong inhibitors of fungal growth; MIC between 0.6 and 1.5 mg/ml as moderated inhibitors and extracts having MIC above 1.6 mg/ml considered as weak inhibitors.Taking in consideration this classification, our extracts shown strong and moderate antifungal activity.

**Table 2:** Minimal Inhibitory Concentrations (MIC) results for antimicrobial activity of extracts from some Cameroonian dietary spices (μg/ml)

**Microorganisms**	**Extracts**							**Antibiotics**	
	**FHF**	**FHB**	**GLB**	**GLL**	**HLR**	**HLBa**	**HLBb**	**Cipro**	**AmphB**
**Bacteria**									
*S. aureus*	-	-	250	500	62.5	500	125	0.25	nd
*S. epidermitis*	-	-	250	500	62.5	500	125	0.25	nd
*E. coli*	500	-	500	-	-	1000	500	0.25	nd
*P. aeruginosa*	500	-	1000	-	-	-	500	1	nd
*MRSA*	-	-		1000	125	500	125	6	nd
*VRE*	-	-		1000	125	1000	125	64	nd
**Fungi**									
*C. albicans*	500	-	500	250	500	nd	500	nd	0.5
*C. krusei*	-	-	1000	250	1000	nd	1000	nd	1
*C. tropicalis*	-	-	125	62.5	125	nd	125	nd	0.5
*A. fumigatus*	500	1000	1000	1000	1000	nd	1000	nd	0.5

FHF: Methanol extract from *Fagara heitzii* fruits; FHB: Methanol extract from *Fagara heitzii* barks; GLB: Methanol extract from *Garcina lucida* barks; GLL: Methanol extract from *Garcina lucida* leaves; HLR: Methanol extract from *Hymenocardia lyrata* roots; HLBa: Aqueous extract from *Hymenocardia lyrata* barks; HLBb: Methanol extract from *Hymenocardia lyrata* barks; Cipro: Ciprofloxacin; Amph B: Amphothericin B; -: MIC**>**1000 μg/mL; nd: not determined.

## Conclusions

It comes out from this study that the methanol extract of *H. lyrata* roots significanly prevent the proliferation of THP-1 and PC-3 cancer cell lines while the methanol extract of *F. heitzii* barks inhibit the growth of Gram-positive bacteria. These extracts could be potentially useful for the development of therapeutic agents against bacteria infections as well as for prostate and leukemia cancer. In addition these results bring supporting data that consumption of plants could reduce our susceptibility to some cancers. These extracts are good candidates for further activity-guided fractionation in the search for new active therapeutic compounds.

## Competing interests

The authors declare that they have no competing interests.

## Authors’ contributions

JPD designed the experiments and wrote the manuscript; SKG and AS participated in the experiments, CAP provided plant material, VK contributed to the manuscript writing process. IAK, SKA and RAV supervised the work. All authors read and approved the final manuscript.

## Pre-publication history

The pre-publication history for this paper can be accessed here:

http://www.biomedcentral.com/1472-6882/13/78/prepub
